# Exosomes from microRNA‐126 overexpressing mesenchymal stem cells promote angiogenesis by targeting the PIK3R2‐mediated PI3K/Akt signalling pathway

**DOI:** 10.1111/jcmm.16192

**Published:** 2020-12-21

**Authors:** Lei Zhang, Pengrong Ouyang, Gaole He, Xiaowei Wang, Defu Song, Yijun Yang, Xijing He

**Affiliations:** ^1^ Department of Orthopaedic Surgery Second Affiliated Hospital of Xi'an Jiaotong University Xi'an Shaanxi Province China; ^2^ Department of Orthopaedic Surgery Xi'an Children's Hospital Xi’an Shaanxi Province China; ^3^ Department of Spine Surgery Hong Hui Hospital Xi’an Jiaotong University Xi’an Shaanxi Province China; ^4^ Xi'an International Medical Center Hospital Xi’an Shaanxi Province China

**Keywords:** angiogenesis, bone marrow mesenchymal stem cells, exosomes, microRNA‐126

## Abstract

microRNA‐126 (miR‐126), an endothelial‐specific miRNA, is associated with vascular homeostasis and angiogenesis. However, the efficiency of miR‐126‐based treatment is partially compromised due to the low efficiency of miRNA delivery *in vivo*. Lately, exosomes have emerged as a natural tool for therapeutic molecule delivery. Herein, we investigated whether exosomes derived from bone marrow mesenchymal stem cells (BMMSCs) can be utilized to deliver miR‐126 to promote angiogenesis. Exosomes were isolated from BMMSCs overexpressed with miR‐126 (Exo‐miR‐126) by ultracentrifugation. *In vitro* study, Exo‐miR‐126 treatment promoted the proliferation, migration and angiogenesis of human umbilical vein endothelial cells (HUVECs). Furthermore, the gene/protein expression of angiogenesis‐related vascular endothelial growth factor (VEGF) and angiotensin‐1 (Ang‐1) were up‐regulated after incubation with Exo‐miR‐126. Additionally, the expression level of phosphoinositol‐3 kinase regulatory subunit 2 (PIK3R2) showed an inverse correlation with miR‐126 in HUVECs. Particularly, the Exo‐miR‐126 treatment contributed to enhanced angiogenesis of HUVECs by targeting PIK3R2 to activate the PI3K/Akt signalling pathway. Similarly, Exo‐miR‐126 administration profoundly increased the number of newly formed capillaries in wound sites and accelerated the wound healing *in vivo*. The results demonstrate that exosomes derived from BMMSCs combined with miR‐126 may be a promising strategy to promote angiogenesis.

## 1 INTRODUCTION

Angiogenesis refers to the generation of new vascular sprouts out of pre‐existing blood vessels. It is a complex multistep process that involves cell proliferation, migration, invasion, and tube formation, as well as maturation and stabilization of newly formed sprouts.^1^ Angiogenesis is an indispensable process throughout the lifetime of humans and is a prerequisite for a wide spectrum of disorder conditions that are characterized by a lack of proper vascular formation, such as heart and brain ischaemia, diabetic foot, poor wound healing, spinal cord injury, myocardial infarction and bone defects.^2^, ^3^ Increasing evidence sustains that neovascularization is mainly mediated by activating endogenous progenitor cells, integrating with exogenous mesenchymal stem cells (MSCs) and/or therapeutic agents such as pro‐angiogenic mRNAs or microRNAs (miRNAs).^4^

miRNAs are a class of endogenous small noncoding RNA molecules (18–25 nucleotides), and based on current understanding, miRNAs regulate cellular processes most likely via posttranscriptional inhibition of gene expression.^5^, ^6^ Lately, increasing evidence has supported the viewpoint that miRNAs play pivotal roles in multiple pathways related to various biological processes, which can assist in regulating a variety of cellular processes and molecular mechanisms.^7^ In particular, certain miRNAs function as pro‐angiogenic regulators.^8^, ^9^, ^10^ Among them, miR‐126, which is specifically expressed in endothelial cells, may be the most important and acts as a key regulator in promoting angiogenesis as well as maintaining vascular integrity. For instance, up‐regulation of miR‐126 can contribute to angiogenesis after vessel injury or hypoxia.^11^ Furthermore, previous studies have found that miR‐126 promotes angiogenesis by down‐regulation the negative regulators of the VEGF pathway, namely, phosphoinositol‐3 kinase regulatory subunit 2 (PIK3R2) and Sprouty‐related EVH1 domain‐containing protein 1 (SPRED1).^11^, ^12^ Additionally, miR‐126 has been proposed to be a biological target that has pro‐angiogenic potential for therapeutics.^13^, ^14^ A better curative effect of diabetic wounds was observed after up‐regulating the miR‐126 expression.^15^ However, the therapeutic efficacy of miR‐126‐based administration is limited due to the low efficiency of miRNA delivery, which remains a major challenge for the use of miRNAs as a therapeutic strategy.^16^ Until recently, overwhelming evidence has demonstrated that miRNAs can be packaged within extracellular vesicles, especially exosomes. Thus, exosomes can serve as therapeutic tools for delivering miRNAs to further exert their biological functions.^17^, ^18^, ^19^

Exosomes, an important form of extracellular vesicles, have been confirmed as the intercellular communication mediators in various physical processes related to cell proliferation and migration, immune modulation and angiogenesis.^20^, ^21^, ^22^ These nano‐sized particles are lipid bilayer structures, with a diameter of approximately ranging from 50 to 200 nm. To date, exosomes have been shown to act as ideal messengers for transferring biological information, such as functional miRNAs, to mediate intercellular communication.^23^ In addition, exosome‐based cell‐free therapy represents an attractive approach due to its several advantages of high stability, low immunogenicity, non‐tumorigenicity and lack of blood‐brain obstructive concern.^24^, ^25^, ^26^ In particular, it has been well established that the exosomes derived from MSCs play a vital role in various physiological processes and diseases.^27^, ^28^, ^29^

Based on the aforementioned findings, we have been suggested that miR‐126, delivered by exosomes derived from bone marrow mesenchymal stem cells (BMMSCs), could promote angiogenesis. Thus, in the current study, we investigated the pro‐angiogenic potential of exosomes from miR‐126 overexpressing BMMSCs (Exo‐miR‐126) in a series of *in vitro* studies and a full‐thickness skin defect model *in vivo*, as well as the underlying molecular mechanisms, in an attempt to provide a potential therapeutic strategy for angiogenesis.

## 2 MATERIALS AND METHODS

### 2.1 Cell culture and treatment

For the present study, human BMMSCs were purchased from Cyagen Biosciences (Guangzhou, China; HUXMA‐01001, https://www.cyagen.com/cn/zh‐cn/product/bone‐marrow‐msc‐HUXMA‐01001.html?from=3#/product‐coa). BMMSCs were cultured in alpha‐minimal essential medium (α‐MEM; Invitrogen, Carlsbad, CA, USA) with 10% foetal bovine serum ((FBS, Gibco, Thermo Fisher Scientific, Waltham, MA, USA) and 1% penicillin‐G/streptomycin (Invitrogen), maintained in a humidified environment (5% CO2) at 37 °C. BMMSCs at passages 3‐6 (P3‐P6) in good growth state were characterized by flow cytometry and multiple differentiation assays, and then used for the downstream research. Flow cytometry was employed to identify cell immunophenotypes, cell suspensions incubated with PBS served as a negative control, and monoclonal antibodies against CD90, CD105, CD45, CD31, CD44, CD146, CD14 and CD34 (eBioscience, CA, USA) were used for the detection. To identify the multiple differentiation potentials, cells were induced to differentiate in osteogenic, adipogenic and chondrogenic differentiation medium separately, followed by Alizarin Red S, Oil Red O and Alcian blue staining, respectively.

Human umbilical vein endothelial cells (HUVECs) were purchased from the American Type Culture Collection (CRL‐1730, https://www.atcc.org/products/all/CRL‐1730.aspx) and cultured in endothelial growth medium‐2 (EGM‐2; Lonza, Walkersville, MD, USA) for following research. After reaching approximately 80% confluence, HUVECs were transfected with miR‐126 mimic (150 nM) and mimic negative control (mimic‐NC), miR‐126 inhibitor (200 nM) and inhibitor negative control (inhibitor‐NC), small interfering RNA (siRNA) targeting PIK3R2 (si‐PIK3R2, 100 nM) and siRNA negative control (si‐NC) (all from Genepharma; Shanghai, China) using Lipofectamine 2000 reagent (Thermo Fisher Scientific) in strict accordance with the manufacturer’s instructions. After transfection for 6 h, the culture medium was changed to fresh medium for the downstream research.

### 2.2 Dual‐luciferase reporter assay

A dual‐luciferase reporter assay was established to verify whether PIK3R2 is the target gene of miR‐126 in HUVECs. In brief, luciferase reporter plasmids (pGL3 vector; Genepharma) with PIK3R2 3′‐UTR containing a mutant or putative binding site of miR‐126 were constructed. HUVECs were transfected with the PIK3R2 3′‐UTR wild‐type (WT) or mutant (MUT) plasmids in the presence of miR‐126 mimic or mimic‐NC in strict accordance with the manufacturer’s specifications. Subsequently, the cells were lysed, followed by quantification of the luciferase activities with a dual‐luciferase reporter assay kit (Yeasen, Shanghai, China).

### 2.3 Production and identification of exosomes

The culture medium was obtained after 48 hours culture of BMMSCs, then exosomes were produced as described previously with minor modifications.^30^ More specifically, the collected supernatant was centrifuged at 300 *g* (10 minutes) and 2000 *g* (30 minutes) to remove dead cells, followed by 10 000 *g* (30 minutes) to discard cellular debris. Then, the obtained supernatant was centrifuged at 100 000 *g* (70 minutes; 70Ti rotor, Beckman Coulter) and washed with phosphate‐buffered saline (PBS; Heart, Xi'an, China) at another 100 000 *g* (70 minutes). After that, the exosome pellet was re‐suspended in PBS for downstream experiments. The obtained exosomes were identified in terms of morphology (transmission electron microscopy, TEM), size (nanoparticle tracking analysis, NTA) and surface markers (Western blotting). For the exosome treatment, to increase the expression of miR‐126, BMMSCs were transfected with miR‐126 mimic (200 nM) or mimic‐NC by Lipofectamine 2000 reagent (Thermo Fisher Scientific). Then, exosomes were extracted from BMMSCs transfected with miR‐126 mimic or mimic‐NC and named Exo‐miR‐126 and Exo‐NC, respectively.

The PKH67 fluorescent labelling kit (Sigma‐Aldrich, St. Louis, MO, USA) was utilized for the exosomes labelling. In brief, the obtained exosomes were re‐suspended in 1 mL diluent C, mixed with 4 µL PKH67, and then incubated together for 4 minutes. The reaction was neutralized by adding 2 mL 5% bovine serum albumin (BSA; Heart), after which the exosomes were washed with PBS at 100,000 g (70 minutes) and then incubated with HUVECs for 4 hours. Subsequently, the cells (HUVECs) were fixed with paraformaldehyde (4%), and the nuclei were labelled with DAPI (0.5 µg/mL; Invitrogen, USA). The images of exosome uptake were captured using a confocal microscope (Carl Zeiss, Oberkochen, Germany).

### 2.4 Exosome treatment

A series of subsequent assays (including proliferation, migration, transwell, tube formation on Matrigel) were conducted to assess the pro‐angiogenic potential of Exo‐miR‐126 on HUVECs. For exosome treatment, HUVECs were divided into the following groups: Control group (treated with an equal volume of PBS); Exo‐NC group (treated with 100 μg/mL Exo‐NC) and Exo‐miR‐126 group (treated with 100 μg/mL Exo‐miR‐126). After treatment for 24 hours, HUVECs were collected for the downstream research.^31^

#### 2.4.1 Proliferation assay

A cell counting kit‐8 assay (CCK‐8 assay) was conducted to evaluate cell viability as described previously.^32^ Briefly, 5 × 10^3^ HUVECs (per well; five replicates per group) were seeded into 96‐well plates (Corning, NY, USA). At a certain time per day, CCK‐8 (10 μL per well) reagent (Dojindo, Kumamoto, Japan) was added to the culture well, and then the mixed sample was incubated for another 2 hours at 37°C. Subsequently, the optical density (OD) value was recorded at a wavelength of 450 nm. All assays were performed in triplicate.

#### 2.4.2 Transwell migration assay

A transwell assay was established to interrogate the migratory ability of HUVECs following previously reported methods.^33^ Briefly, 2 × 10^4^ HUVECs were re‐suspended in 200 μL serum‐free medium and plated into the apical chamber of transwell plates (24‐well; pore size: 8 μm; Corning), while 700 μL culture medium containing 20% FBS was added into the basolateral chamber. After incubating for 10 hours, the non‐migrated cells were wiped off with a cotton swab, while the migrated cells on the lower side of the membrane were fixed with 4% paraformaldehyde (20 minutes) and then stained with crystal violet (0.5%) for 20 minutes. After washing three times with PBS, the migrated cells were observed and imaged using an inverted microscope (Olympus, Tokyo, Japan), and the number of migrated cells from five randomly selected fields was calculated for each group. All assays were performed in triplicate.

#### 2.4.3 Scratch wound healing assay

A wound healing assay was performed as previously reported methods.^20^ In brief, 2 × 10^5^ (per well) HUVECs were plated into culture plates (12‐well; Corning) and incubated till they reached 90% confluence. After that, a sterile 200‐μL pipette tip was utilized to make a scratch on the cell monolayer, and the floating cells were removed by gently washing with PBS. Then, the cells were incubated for another 24 hours. Images were captured at 0 hour, 12 hours and 24 hours post‐wounding, respectively. All assays were performed in triplicate, and the outcomes were analysed using ImageJ software.

#### 2.4.4 Tube formation assay

For the tube formation assay on Matrigel, 50 μL of cold Matrigel (Corning) was plated in each culture well (96‐well plates) and incubated for 30 minutes at 37°C. After that, 2 × 10^4^ HUVECs were seeded in each well (five replicates per group) and incubated for 6 hours. Subsequently, tube‐like structures were photographed using a microscope (Olympus), and the indicators of vessel formation in terms of total branching points and total tube length were analysed using ImageJ software. All assays were performed in triplicate.

### 2.5 Quantitative real‐time PCR (qRT‐PCR) analysis

Total RNA was extracted using TRIzol reagent (Invitrogen, CA, USA), and the miRNA expression was quantified using TaqMan® miRNA assays (Applied Biosystems, CA, USA). After RNA was reverse‐transcribed to cDNA with the PrimeScript RT reagent Kit (TaKaRa, Dalian, China), qRT‐PCR analysis was conducted with the SYBR® Premix Dimer Eraser kit (Takara). U6 was utilized as the internal reference for miR‐126, while β‐actin was used as the internal reference for other target genes. The qRT‐PCR primer sequences were as follows: miR‐126, forward 5'‐GCTGTCAGTTTGTCAAATA‐3' and reverse 5'‐GTGCAGGGTCCGAGGT‐3'; U6, forward 5'‐CTCGCTTCGGCAGCACA‐3' and reverse 5'‐AACGCTTCACGAATTTGCGT‐3'; VEGF, forward 5'‐CATCCAATCGAGACCCTGGTG‐3' and reverse 5'‐ TTGGTGAGGTTTGATCCGCATA‐3'; Ang‐1, forward 5'‐GAAGGGAACCGAGCCTATTC‐3' and reverse 5'‐AGGGCACATTTGCACATACA‐3'; PIK3R2, forward 5'‐GCACCACGAGGAACGCACTT‐3' and reverse 5'‐CGTCCACTACCACGGAGCAG‐3'; and β‐actin forward 5'‐TGGCACCCAGCACAATGAA‐3' and reverse 5'‐ CTAAGTCATAGTCCGCCTAGAAGCA‐3'.

### 2.6 Western blot analysis

Western blotting was performed as described previously with minor modifications.^34^ In brief, HUVEC or exosomal proteins were extracted and incubated overnight at 4°C with primary antibodies including anti‐CD9 (1:1000; Cell Signaling Technology, Danvers, MA, USA; #13174), anti‐CD81 (1:1000; Abcam, Cambridge, Britain; ab109201), anti‐ALIX (1:1000; Cell Signaling Technology; #2171), anti‐VEGF (1:1000; Abcam; ab46154), anti‐Ang‐1 (1:500; Abcam; ab8451), anti‐PIK3R2 (1:1000; Abcam; ab131067), anti‐phosphorylated Akt (p‐Akt; 1:1000, Cell Signaling Technology; #4060), anti‐Akt (1:1000, Cell Signaling Technology; #4685) and anti‐β‐actin (1:2000; Proteintech, Rosemont, USA; #60008‐1‐lg). After incubation with horseradish peroxidase‐conjugated secondary antibodies (1:5000, ProteinTech; SA00001‐1 or SA00001‐2) at room temperature for 2 hours, the blots were visualized using chemiluminescence substrate (ECL kit; Beyotime, Shanghai, China) and the quantification of protein bands was analysed with ImageJ software. β‐actin was used as the loading control for internal normalization.

### 2.7 Mouse skin wound model and treatments

It is well acknowledged that angiogenesis plays a crucial role in wound healing, thus, a mouse skin defect model was established to examine the pro‐angiogenic behaviour of Exo‐miR‐126 *in vivo*.^32^ All protocols were approved by the Animal Research Committee of Xi'an Jiaotong University. For the research, 24 male C57BL/6 mice (8‐week‐old, weighing 20–25 g) were shaved and anaesthetized before operation according to the animal protocol, and then a full‐thickness excisional skin wound was created on the dorsum. After that, the mice were randomly divided into the following groups : (1) Control group (100 μL PBS, n = 8); (2) Exo‐NC group (200 μg Exo‐NC in 100 μL PBS, n = 8); and (3) Exo‐miR‐126 group (200 μg Exo‐miR‐126 in 100 μL PBS, n = 8). Subsequently, exosomes or PBS only were subcutaneously injected into 4 sites (25 μL per site) around the wounds.

### 2.8 Histological and immunofluorescence analyses

Following local administration of exosomes, the wounds were photographed at the scheduled time points (0, 4, 9, 14 days post‐operation), and the wound closure area was analysed using ImagJ software to evaluate the healing rate. 14 days after surgery, the mice were killed, skin specimens were collected and then analysed using histopathological methods.^32^ More specifically, the harvested skin samples (including the defect epicentre and the surrounding healthy skin) were fixed in 4% paraformaldehyde (Heart), then dehydrated using ethanol. After embedding in paraffin, the specimens were cut into 10‐μm‐thickness, subjected to haematoxylin and eosin (H&E) staining. Additionally, to observe the newborn capillaries in skin defect sections, immunofluorescence staining against CD31 and CD34 was performed.^35^ Initially, the obtained tissues were fixed in 4% paraformaldehyde solution at 4°C overnight, then dehydrated with 30% sucrose solution and embedded in OCT. After that, the obtained sections (10‐μm thick) were incubated with primary antibodies against CD34 (Abcam,1:100) and CD31 (Abcam,1:100) at 4°C overnight, and then incubated with secondary antibodies (1:300 or 1:400, Servicebio; Wuhan, China), respectively, at room temperature for 1 hour. Image‐Pro Plus 6 software was used for quantitative analysis.

### 2.9 Statistical analysis

The measurement data are presented as mean ± standard deviation (SD), and all assays were performed at least three times. Differences between groups were assessed by Student's *t* test or one‐way analysis of variance (ANOVA), with Tukey's post hoc analysis conducted for post hoc test. *P* < 0.05 was considered to be statistically significant.

## 3 RESULTS

### 3.1 Identification of BMMSC‐derived exosomes

The results of flow cytometry analysis revealed that the BMMSCs were positive for CD105, CD90, CD44 and CD146, while negative for CD45 and CD31 CD34 and CD14 (Figure S1A). In addition, the BMMSCs had the potential to osteogenic (as evidenced by Alizarin Red S staining; left), adipogenic (as evidenced by Oil Red O staining; middle) and chondrogenic (as evidenced by Alcian blue staining; left) differentiation when cultured in the respective culture medium (Figure S1B).

**FIGURE 1:**
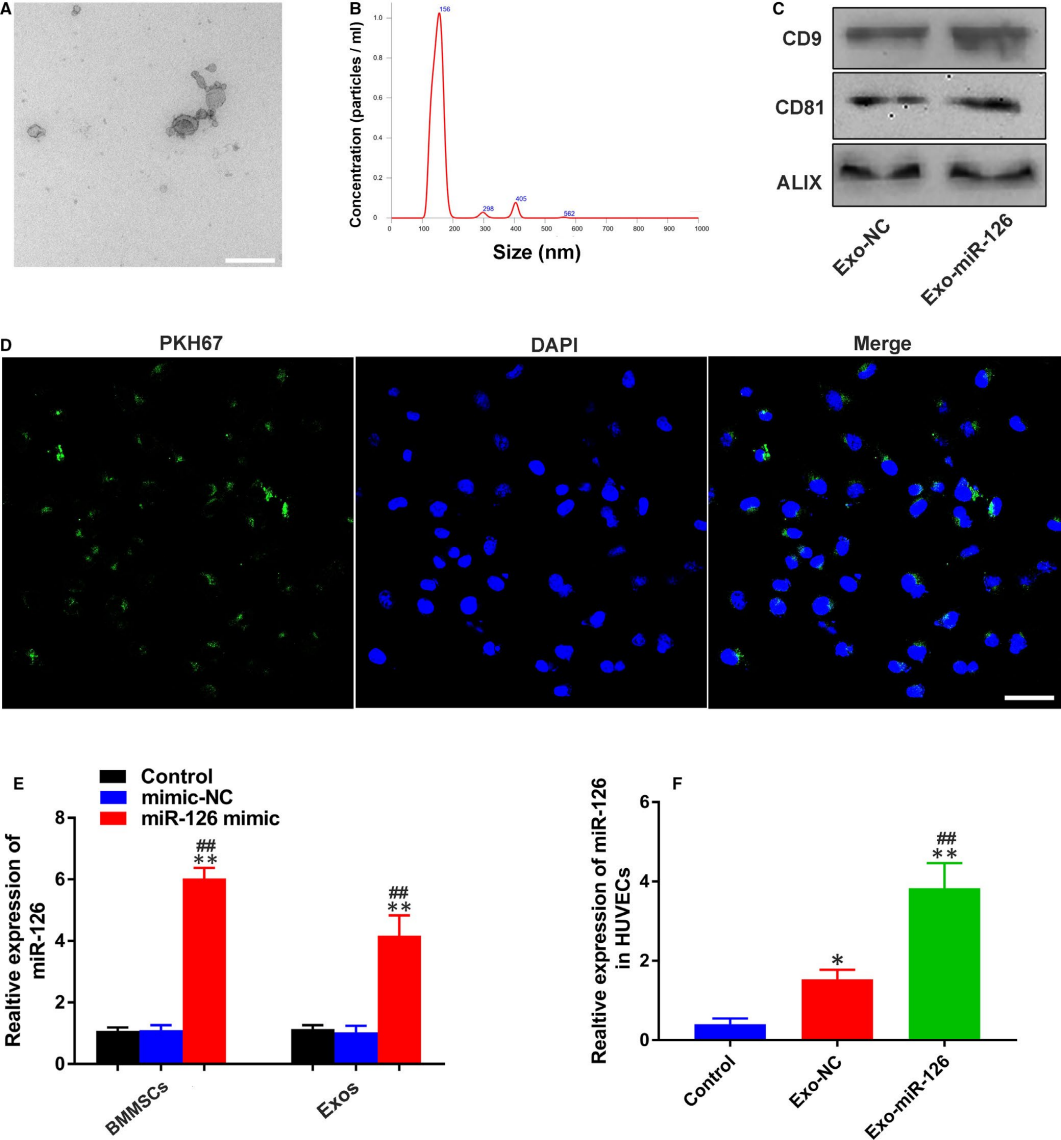
Identification and internalization of exosomes. (A) Morphology of exosomes under transmission electron microscopy (scale bar: 100 nm). (B) Size of exosomes identified by nanoparticle tracking analysis. (C) Exosomal surface markers CD9, CD81 and ALIX detected by Western blotting. (D) Fluorescence microscopy analysis of PKH67‐labelled exosomes (green) internalized by HUVECs. Nuclei were stained with DAPI (blue) for counterstaining (scale bar: 50 μm). (E) miR‐126 expression in BMMSCs and BMMSC‐derived exosomes detected by qRT‐PCR (n = 3), **P < 0.01 vs. Control group, ^##^ *P* < 0.01 vs. mimic‐NC group (F) miR‐126 expression in HUVECs treated with PBS, Exo‐NC and Exo‐miR‐126 detected by qRT‐PCR (n = 3). *P < 0.05, **P < 0.01 vs. Control group, ^##^ *P* < 0.01 vs. Exo‐NC group. HUVECs, human umbilical vein endothelial cells; Exo‐NC, exosomes derived from BMMSCs transfected with NC‐mimic; Exo‐miR‐126, exosomes derived from microRNA‐126 overexpressing bone marrow mesenchymal stem cells

Exosomes were extracted from the supernatant of BMMSCs by ultracentrifugation, and then a series of assays were carried out to verify their identification. TEM analysis showed that the obtained nanoparticles had sphere‐ or cup‐shaped morphology (Figure 1A), which was consist with previous findings.^30^ Meanwhile, nanoparticle analysis was performed to analyse the particle size, and the obtained results demonstrated that the diameters of these nanoparticles mainly ranged from 30 to 200 nm (Figure 1B), coinciding with the size of exosomes.^36^ As for Western blot analysis, positive exosomal protein markers including CD9, CD81 and ALIX were observed in these nano‐vesicles (Figure 1C), further confirming their identity as exosomes. Additionally, The PKH67 kit was used to confirm cellular exosome uptake, based on the analysis of confocal laser microscopy, HUVECs were stained positively for PKH67 (green), which confirmed that the uptake of exosomes was successful (Figure 1D). Subsequently, qRT‐PCR analysis was carried out to evaluate the miR‐126 expression in exosomes and BMMSCs after transfection, and the obtained results revealed that the miR‐126 expression levels in BMMSCs transfected with miR‐126 mimic and their secreted exosomes (Exo‐miR‐126) were significantly higher than those of BMMSCs transfected with mimic‐NC and Control group and their secreted exosomes, respectively (*P* < 0.01; Figure 1E). Additionally, the miR‐126 expression in HUVECs was significantly up‐regulated after Exo‐miR‐126 treatment relative to Exo‐NC and Control groups (*P* < 0.01; Figure 1F).

### 3.2 Exo‐miR‐126 enhanced the proliferation, migration and tube formation of HUVECs in vitro

**FIGURE 2:**
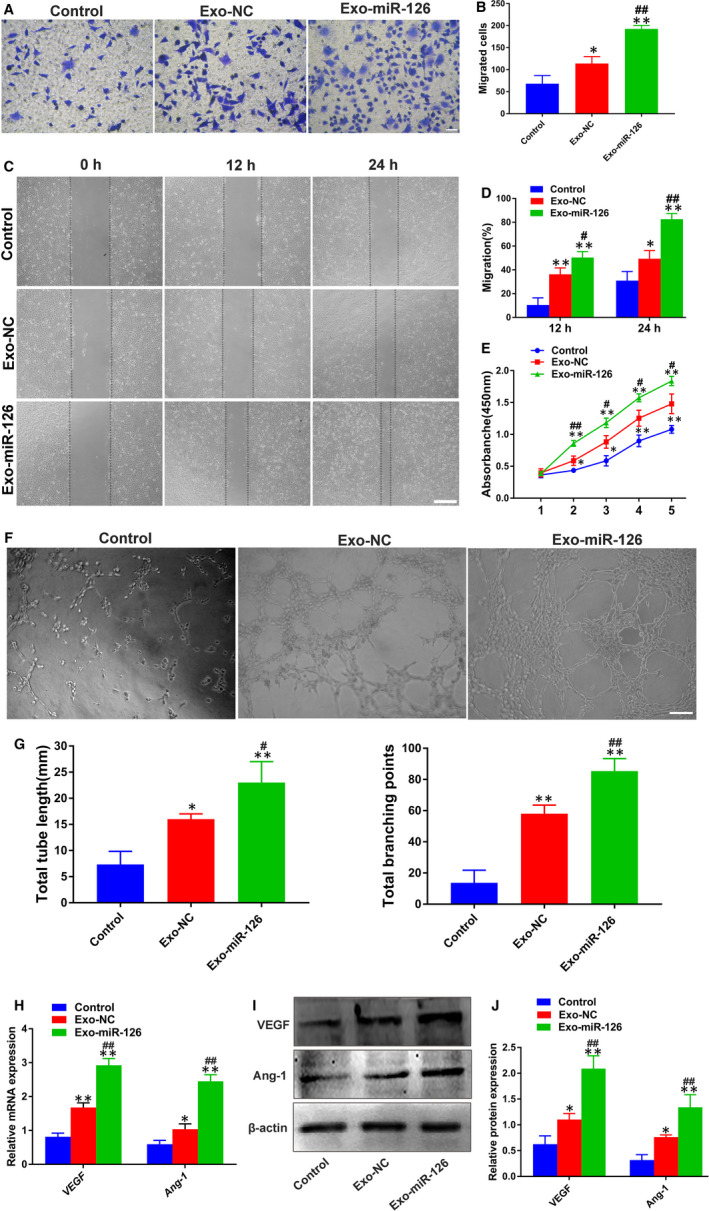
Exo‐miR‐126 potentiated the proliferation, migration and angiogenic activities of HUVECs *in vitro*. (A) The migration of HUVECs treated with PBS, Exo‐NC and Exo‐miR‐126 was detected by transwell assay (scale bar: 100 μm). (B) Quantitative analysis of the migrated cells in (A) (n = 3). (C) Representative images of scratch wound assay in HUVECs treated with PBS, Exo‐NC and Exo‐miR‐126 (scale bar: 200 μm). (D) Quantitative analysis of the migration rates in (C) (n = 3). (E) The proliferation of HUVECs exposed to PBS, Exo‐NC and Exo‐miR‐126 was tested by CCK‐8 assay (n = 3). (F) Representative images of tube formation assay in HUVECs receiving different treatments (scale bar: 200 μm). (G) Quantitative analyses of the total tube length and total branching points in (F) (n = 5). (H) The pro‐angiogenic genes *VEGF* and *Ang‐1* in HUVECs were analysed by qRT‐PCR (n = 3). (I) Detection of the protein levels of VEGF and Ang‐1 in HUVECs by Western blot analysis. (J) Quantitative analysis of the relative protein expression in (I) (n = 3). *P < 0.05, **P < 0.01 vs. Control group, **^#^** *P* < 0.05, **^##^** *P* < 0.01 vs. Exo‐NC group. HUVECs, human umbilical vein endothelial cells; Exo‐NC, exosomes derived from BMMSCs transfected with NC‐mimic; Exo‐miR‐126, exosomes derived from microRNA‐126 overexpressing bone marrow mesenchymal stem cells; CCK‐8 assay, cell counting kit‐8 assay. VEGF, vascular endothelial growth factor; Ang‐1, angiotensin‐1

Further, a series of functional assays were established to explore the pro‐angiogenic potential of Exo‐miR‐126. Based on the results of transwell assay, HUVECs in the Exo‐miR‐126 group demonstrated a stronger migration ability than those in Exo‐NC and Control groups (*P* < 0.01; Figure 2A and B). Also, the scratch wound healing assay revealed that the migration ability of HUVECs in the Exo‐miR‐126 group was enhanced relative to that of the other two groups (*P* < 0.05 or *P* < 0.01; Figure 2C and D), although the cells in Exo‐NC group exhibited enhanced migration ability than that of Control group (*P* < 0.05 or *P* < 0.01; Figure 2C and D). Additionally, cells incubated with either Exo‐miR‐126 or Exo‐NC both exhibited a significantly augmented proliferation rate compared with Control group (*P* < 0.05 or *P* < 0.01; Figure 2E), Besides, the cells in Exo‐miR‐126 group demonstrated a higher proliferation rate than that in Exo‐NC group (*P* < 0.05 or *P* < 0.01; Figure 2E).

**FIGURE 3:**
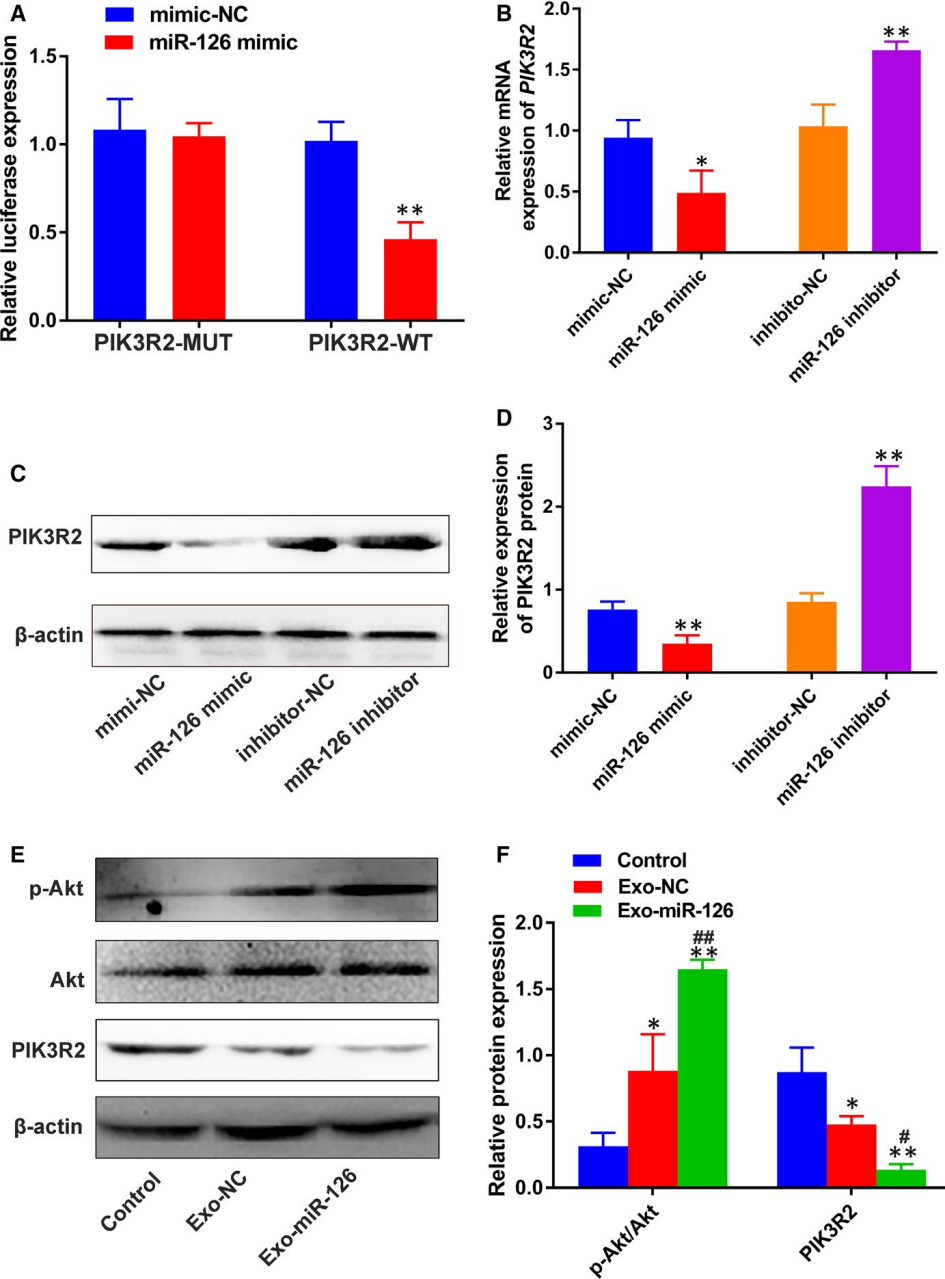
miR‐126 activated the PI3K/Akt signalling pathway via down‐regulation of PIK3R2. (A) The relative luciferase activity of luciferase reports with wild‐type or mutant PIK3R2 was determined in HUVECs, transfected with mimic‐NC or miR‐126 mimic (n = 3). **P < 0.01 vs. mimic‐NC group. (B) The relative mRNA expression of *PIK3R2* in HUVECs transfected with miR‐126 mimic or miR‐126 inhibitor detected by qRT‐PCR (n = 3). *P < 0.05 vs. mimic‐NC group, **P < 0.01 vs. inhibitor‐NC group. (C) PIK3R2 protein expression in HUVECs transfected with miR‐126 mimic or miR‐126 inhibitor measured by Western blotting. (D) Quantitative analysis of the relative protein expression in (C) (n = 3). **P < 0.01 vs. mimic‐NC group or inhibitor‐NC group. (E) PIK3R2, Akt and p‐Akt protein levels in HUVECs incubated with PBS, Exo‐NC and Exo‐miR‐126 measured using Western blotting. (F) Quantitative analysis of the relative protein expression in (E) (n = 3). *P < 0.05, **P < 0.01 vs. Control group, **^#^** *P* < 0.05, **^##^** *P* < 0.01 vs. Exo‐NC group. HUVECs, human umbilical vein endothelial cells; PIK3R2, phosphoinositol‐3 kinase regulatory subunit 2; Exo‐NC, exosomes derived from BMMSCs transfected with NC‐mimic; Exo‐miR‐126, exosomes derived from microRNA‐126 overexpressing bone marrow mesenchymal stem cells

More importantly, as shown in Figure 2F, both the cells in Exo‐miR‐126 and Exo‐NC group showed stronger tube formation ability when compared with Control group based on the quantitative analyses (*P* < 0.05 or *P* < 0.01; Figure 2G), Besides, the angiogenesis potential of HUVECs was significantly enhanced after Exo‐miR‐126 treatment than Exo‐NC (*P* < 0.05 or *P* < 0.01; Figure 3G). Afterwards, qRT‐PCR and Western blot analysis were conducted to detect the gene and protein expression of VEGF and Ang‐1, which are considered critical molecules for vessel formation and stability.^37^, ^38^ As shown in Figure 2H, HUVECs in the Exo‐miR‐126 and Exo‐NC groups had significantly elevated expression levels of genes (*VEGF* and *Ang‐1*) than the Control group (*P* < 0.05 or *P* < 0.01), and the cells incubated with Exo‐miR‐126 demonstrated a higher expression level relative to Exo‐NC group (*P* < 0.01). In line with these findings, Western blotting showed that HUVECs in Exo‐miR‐126 group possessed higher protein levels of VEGF and Ang‐1 than the other two groups (*P* < 0.01; Figure 2I and J). These results collectively indicated that Exo‐miR‐126 can promote the proliferation, migration and angiogenesis of HUVECs *in vitro*.

### 3.3 miR‐126 down‐regulated PIK3R2 to activate the PI3K/Akt signalling pathway in HUVECs

PIK3R2 is a potential target of miR‐126, and thus, a dual‐luciferase assay was carried out to further verify its accuracy in HUVECs. As shown in Figure 3A, compared to mimic‐NC group, miR‐126 mimic significantly decreased the intensity of luciferase activity in the PIK3R2‐WT group (*P* < 0.01), while, no significant difference was observed in the PIK3R2‐MUT group (*P* > 0.05), highlighting the specific binding of miR‐126 to PIK3R2 in HUVECs. Then, the expression of PIK3R2 was detected in HUVECs after incubation with miR‐126 mimic or miR‐126 inhibitor. As shown in Figure 3B, there was a significant decrease in the expression of *PIK3R2* after treatment with miR‐126 mimic than mimic‐NC (*P* < 0.05), while reverse effects were observed in the miR‐126 inhibitor group (*P* < 0.01). Moreover, according to the results and quantitative analysis of Western blotting (Figure 3C and D **)**, the protein level of PIK3R2 was down‐regulated after incubating with miR‐126 mimic relative to that of mimic‐NC (*P* < 0.01). In contrast, the protein level of PIK3R2 was up‐regulated in the miR‐126 inhibitor group compared with that of inhibitor‐NC group (*P* < 0.01), confirming that miR‐126 can suppress PIK3R2 in HUVECs.

Then, the expression of PIK3R2 was detected in HUVECs after incubation with Exo‐miR‐126. Based on the results and quantitative analysis of Western blotting (Figure 3E and F), the PIK3R2 protein level was down‐regulated after incubation with Exo‐miR‐126 compared to that of Exo‐NC and control groups (*P* < 0.05 or *P* < 0.01). It has been reported that miR‐126 can mediate the angiogenesis process by targeting PIK3R2 to activate the PI3K/Akt signalling pathway.^12^ To further verify whether BMMSC‐derived exosomes could deliver miR‐126 to activate this pathway, Western blotting was carried out to determine the protein levels of Akt and p‐Akt in HUVECs following incubation with exosomes. As shown in Figure 3E and F, the ratio of p‐Akt/Akt was increased in Exo‐miR‐126 group than that in Control and Exo‐NC groups (*P* < 0.01).

**FIGURE 4:**
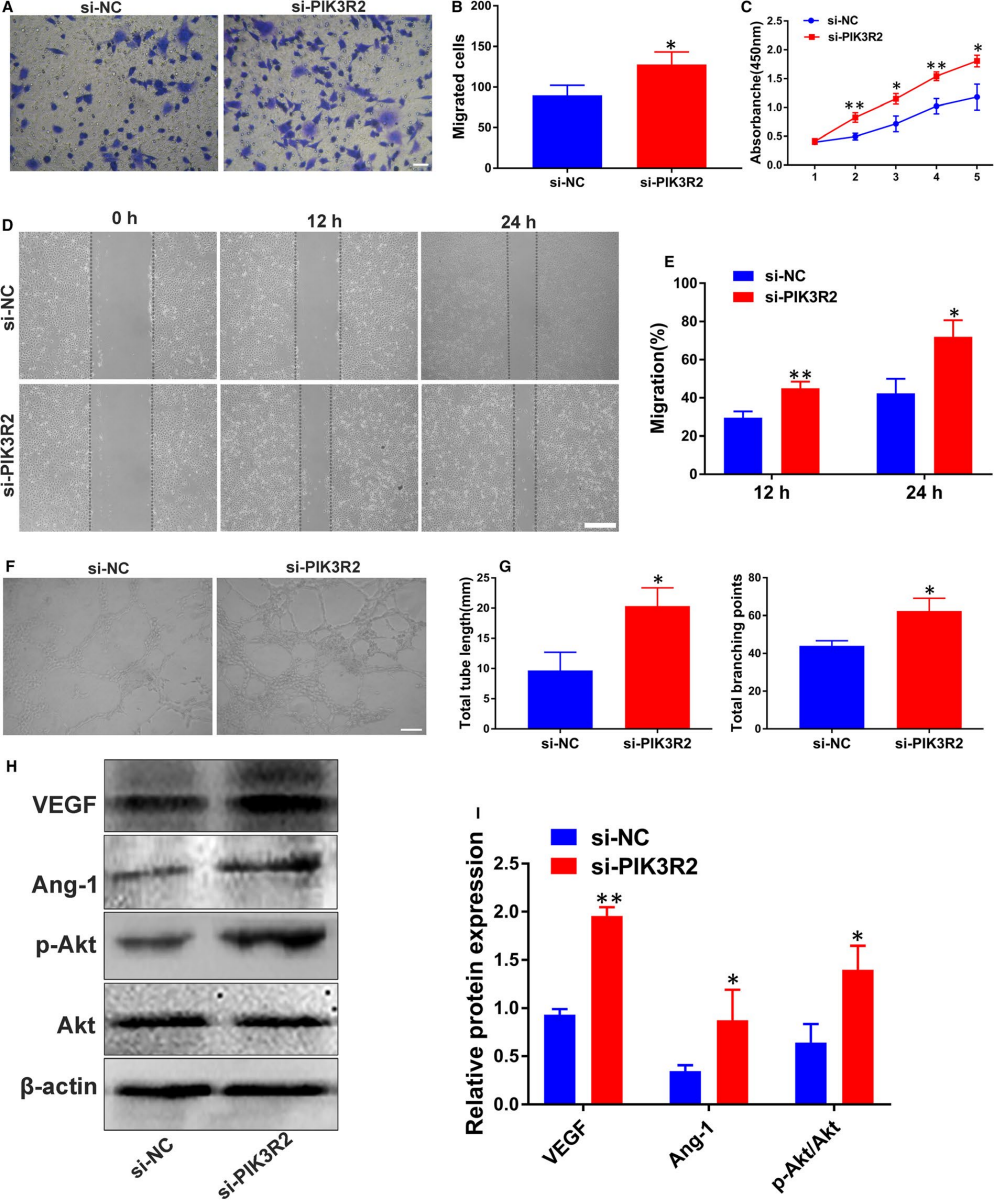
Involvement of PIK3R2 in the role of miR‐126 in HUVECs. (A) The migration of HUVECs stimulated with si‐NC and si‐PIK3R2 was detected by transwell assay (scale bar: 100 μm). (B) Quantitative analysis of the migrated cells in (A) (n = 3). (C) The proliferation of HUVECs exposed to si‐NC and si‐PIK3R2 was tested by CCK‐8 assay (n = 3). (D) Representative images of scratch wound assay in HUVECs treated with si‐NC and si‐PIK3R2 (scale bar: 200 μm). (E) Quantitative analysis of the migration rates in (D) (n = 3). (F) Representative images of tube formation assay in HUVECs treated with si‐NC and si‐PIK3R2 (scale bar: 200 μm). (G) Quantitative analysis of the total tube length and total branching points in (F) (n = 5). (H) Detection of the protein levels of VEGF, Ang‐1, p‐Akt and Akt in HUVECs by Western blot analysis. (I) Quantitative analysis of the relative protein expression in (H) (n = 3). *P < 0.05, **P < 0.01 vs. si‐NC group. HUVECs, human umbilical vein endothelial cells; CCK‐8 assay, cell counting kit‐8 assay; PIK3R2, phosphoinositol‐3 kinase regulatory subunit 2; VEGF, vascular endothelial growth factor; Ang‐1, angiotensin‐1

Additionally, the involvement of PIK3R2 in HUVECs was also investigated to determine the role of miR‐126. To this end, cell proliferation, migration and tube formation assays were performed after HUVECs were treated with either si‐NC or si‐PIK3R2. The obtained data showed that HUVECs in the si‐PIK3R2 group exhibited an enhanced migration ability and higher proliferation rate compared with si‐NC group (*P* < 0.05 or *P* < 0.01; Figure 4A‐E). Moreover, after treatment with si‐PIK3R2, the HUVECs demonstrated a higher potential of forming capillary‐like structures based on the analyses of total tube length and total branching points (*P* < 0.05; Figure 4F and G). Additionally, the results of Western blot analysis showed that the decrease of PIK3R2 resulted in up‐regulated protein levels of VEGF and Ang‐1 (*P* < 0.05 or *P* < 0.01; Figure 4H and I). Notably, the ratio of p‐Akt/Akt was significantly increased following si‐PIK3R2 administration than si‐NC (*P* < 0.05; Figure 4I). These results collectively suggested that miR‐126‐mediated angiogenesis by down‐regulating PIK3R2 to activate the PI3K/Akt signalling pathway.

### 3.4 Exo‐miR‐126 accelerated cutaneous wound healing in vivo

**FIGURE 5:**
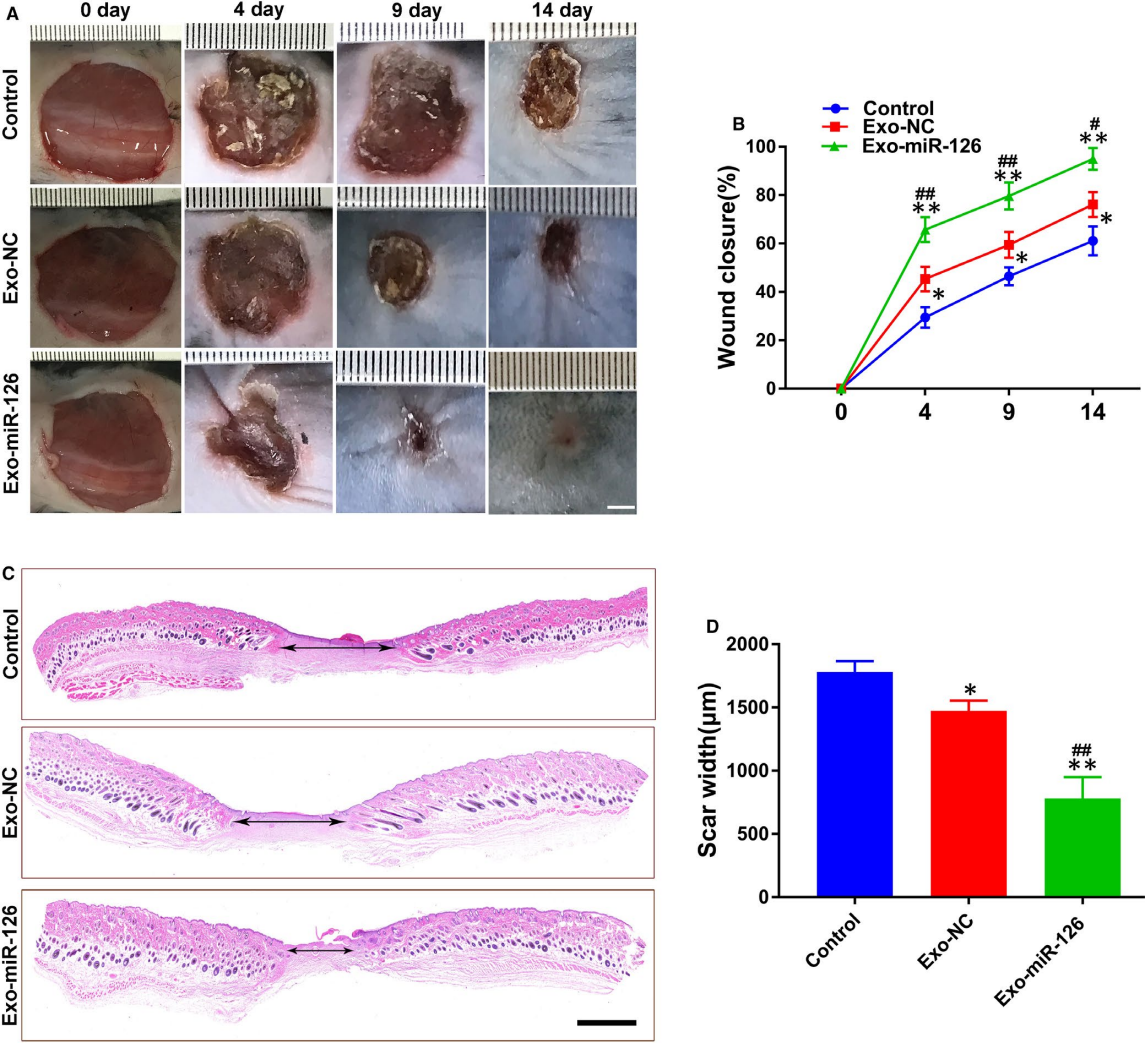
Exo‐miR‐126 accelerated cutaneous wound healing in mice. (A) Gross view of wounds in mice receiving different treatments at days 4, 9 and 14 post‐wounding (scale bar: 2 mm). (B) The rate of wound closure in wounds receiving different treatments at the indicated times (n = 8). (C) H&E staining of wound sections from mice receiving different treatments at 14 days after operation. The double‐headed black arrows indicate the edges of the scars (scale bar: 500 μm). (D) Quantitative analysis of scar widths in (C) (n = 8). *P < 0.05, **P < 0.01 vs. Control group, **^#^** *P* < 0.05, **^##^** *P* < 0.01 vs. Exo‐NC group. Exo‐NC, exosomes derived from BMMSCs transfected with NC‐mimic; Exo‐miR‐126, exosomes derived from microRNA‐126 overexpressing bone marrow mesenchymal stem cells

Based on the foregoing results, a full‐thickness cutaneous wound model was created to evaluate the effects of Exo‐miR‐126 on newly generated capillaries and on wound healing. Exosomes or PBS only were subcutaneously injected into 4 sites (25 μL per site) around the wounds every three days (on days 0, 3, 6, 9 and 12 post‐surgery) based on a pre‐study analysis of the retention of the administered exosomes in skin tissues (Figure S2). All the mice were taken good care in strict accordance with the animal protocols. As shown in Figure 5A, after local injection of exosomes or an equal volume of PBS, the wound closure rate in Exo‐miR‐126 and Exo‐NC treated mice was significantly increased when compared with Control group (*P* < 0.05 or *P* < 0.01; Figure 5B), more importantly, Exo‐miR‐126 group exhibited a significantly higher rate than Exo‐NC group (*P* < 0.05 or *P* < 0.01; Figure 5B). Also, the outcomes of H&E staining and quantitative analysis showed that Exo‐miR‐126 treated wounds had a lower level of scar formation than that of the Exo‐NC and Control groups (*P* < 0.01; Figure 5C and D), although the Exo‐NC group also showed a significantly reduced level of scar formation relative to the Control group (*P* < 0.05; Figure 5C and D).

### 3.5 Exo‐miR‐126 enhanced angiogenesis in the wound sites

**FIGURE 6:**
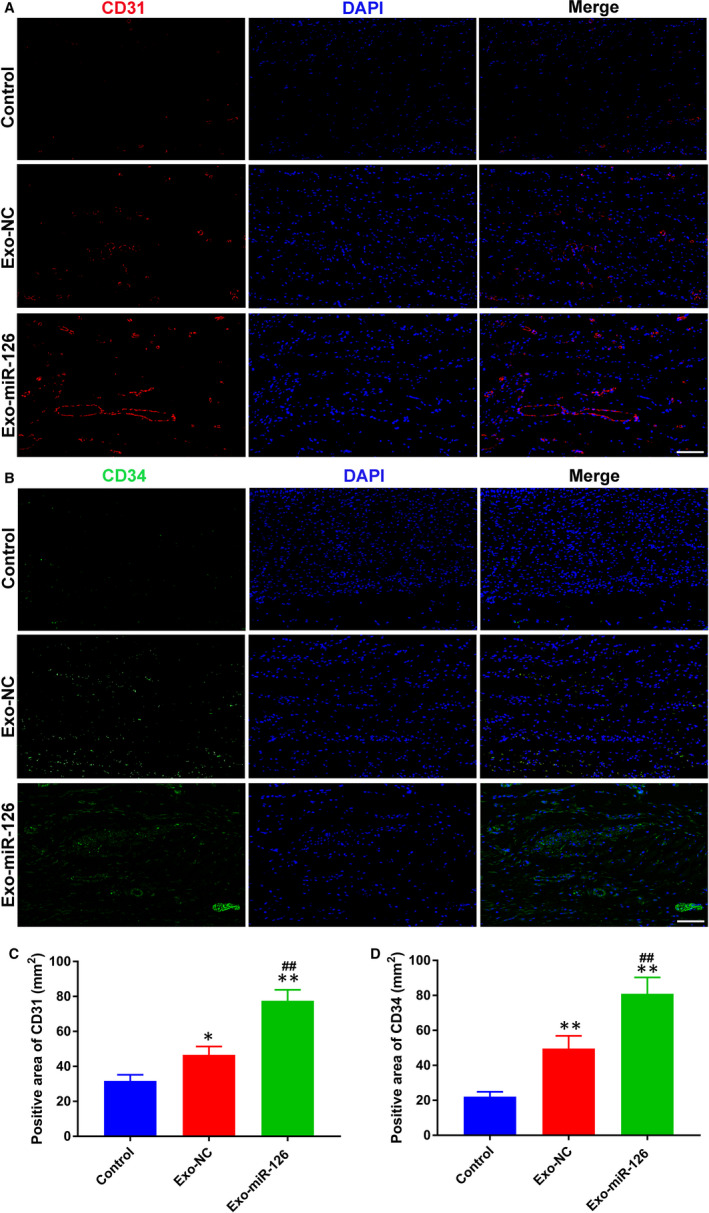
Exo‐miR‐126 enhanced angiogenesis in the wound sites of mice. (A) CD31 immunofluorescence staining of wound sections from mice receiving different treatments at day 14 post‐wounding (scale bar: 50 μm). (B) Representative images of CD34 staining of wound sections from mice receiving different treatments at day 14 post‐wounding (scale bar: 50 μm). (C) Quantitative analysis of the CD31‐positive area in (A) (n = 8). (D) Quantitative analysis of the CD34‐positive area in (B) (n = 8). *P < 0.05, **P < 0.01 vs. Control group, **^##^** *P* < 0.01 vs. Exo‐NC group. Exo‐NC, exosomes derived from BMMSCs transfected with NC‐mimic; Exo‐miR‐126, exosomes derived from microRNA‐126 overexpressing bone marrow mesenchymal stem cells

Immunofluorescence staining for the endothelial markers CD31 and CD34 was conducted to identify the newborn vessels. As shown in Figure 6A, a larger amount of blood vessels staining for CD31 were observed in Exo‐miR‐126 group when compared with the Exo‐NC and Control groups (*P* < 0.01; Figure 6A and C), though more newly generated vessels also observed in Exo‐NC relative to Control group, showing statistical significance (*P* < 0.05; Figure 6C). Meanwhile, CD34 expression in the wound sites was quantified to further confirm the potential of Exo‐miR‐126 to promote blood vessel formation (Figure 6B). In line with the outcomes of CD31 analysis, compared with Control group, the vessel number in Exo‐NC group was significantly higher as determined by CD34 immunofluorescence staining (*P* < 0.01; Figure 6D). Moreover, the results of analysis indicated that the Exo‐miR‐126 group had more newly formed capillaries than Exo‐NC group (*P* < 0.01; Figure 6D).

## 4 DISCUSSION

In the current study, we found that exosomes derived from miR‐126 overexpressing BMMSCs significantly promoted the proliferation, migration and tube formation of HUVECs *in vitro* by transferring miR‐126, which in turn suppressed PIK3R2 to activate the PI3K/Akt signalling pathway. Additionally, local administration of Exo‐miR‐126 profoundly enhanced neo‐angiogenesis in the defect zone and accelerated the wound healing *in vivo*. These findings suggest that BMMSC‐derived exosomes combined with miR‐126 may represent a promising strategy for angiogenesis.

Angiogenesis involves a series of activities including cell proliferation, migration and tubular network formation, which provides sufficient oxygen, nutrients and growth factors to the target tissues, for example, to the wound sites, further facilitating their repair process.^32^, ^39^ The cellular mechanisms of neo‐angiogenesis are complicated and involve multiple signalling pathways. Lately, increasing and overwhelming evidence has suggested that a group of miRNAs plays vital roles in the angiogenic process.^40^ Thus, regulation of specific miRNAs may reveal novel avenues for neovascularization related to regenerative medicine. However, the instability of miRNAs in extracellular circulation poses a critical challenge, as miRNAs must be transported to target tissues by efficient delivery systems.^16^ Recently, exosome‐based cell‐free delivery systems have emerged as promising vectors to transfer miRNAs.^17^, ^18^, ^19^

It has been well established that intercellular communication occurs directly via gap junctions or indirectly via soluble factors (such as cytokines, growth factors, chemokines) and extracellular vesicles (ectosomes, exosomes).^23^ In particular, the exosomes among these secreted vesicles act as messengers that transfer certain molecular elements (such as proteins, mRNAs, lipids, miRNAs) to mediate the therapeutic efficacy of their donor cells in several tissue regeneration scenarios. Based on current understanding, regulating the miRNA expression in exosomes can significantly promote angiogenesis.^41^, ^42^ All these studies suggest that exosomes act as natural therapeutic nano‐delivery tools and play vital roles in angiogenesis.^28^, ^42^, ^43^ In addition, MSC exosomes‐based therapies overcome the risks and obstacles associated with stem cell transplantation approaches and possess a lower risk of aneuploidy and lower propensity to trigger immune rejection.^44^ More importantly, miRNAs can be easily encapsulated into exosomes and these exosomes possess higher delivery efficiency.^45^

In the current study, we found that miR‐126 expression was highly increased in exosomes secreted from BMMSCs transfected with miR‐126 mimic. Then, BMMSC‐derived exosomes acted as a natural vehicle and delivered miR‐126 into HUVECs effectively, thereby promoting cell proliferation, migration and tube formation. miR‐126 is an endothelial cell‐specific miRNA that plays a pivotal role in neovascularization. Knockout of miR‐126 in mice caused leaky vessels, haemorrhaging, oedema and partial embryonic lethality due to loss of vascular integrity and defective angiogenesis.^46^ In a case of myocardial infarction, Danhong injection contributed to post‐infarct angiogenesis mainly by up‐regulating miR‐126 expression to activate the ERK/VEGF pathway.^47^ A recent study demonstrated that the HOTAIR/miR‐126/SCEL pathway could be regulated to contribute to burnt wound healing, as miR‐126 mediates angiogenesis by promoting endothelial cell proliferation, migration and angiogenesis.^48^ Having demonstrated the angiogenesis‐inducing potential of Exo‐miR‐126 on HUVECs, we also investigated the gene and protein expression of the angiogenesis‐related molecules VEGF and Ang‐1, which were both up‐regulated following Exo‐miR‐126 treatment, further confirming the pro‐angiogenic potential of Exo‐miR‐126.

**FIGURE 7:**
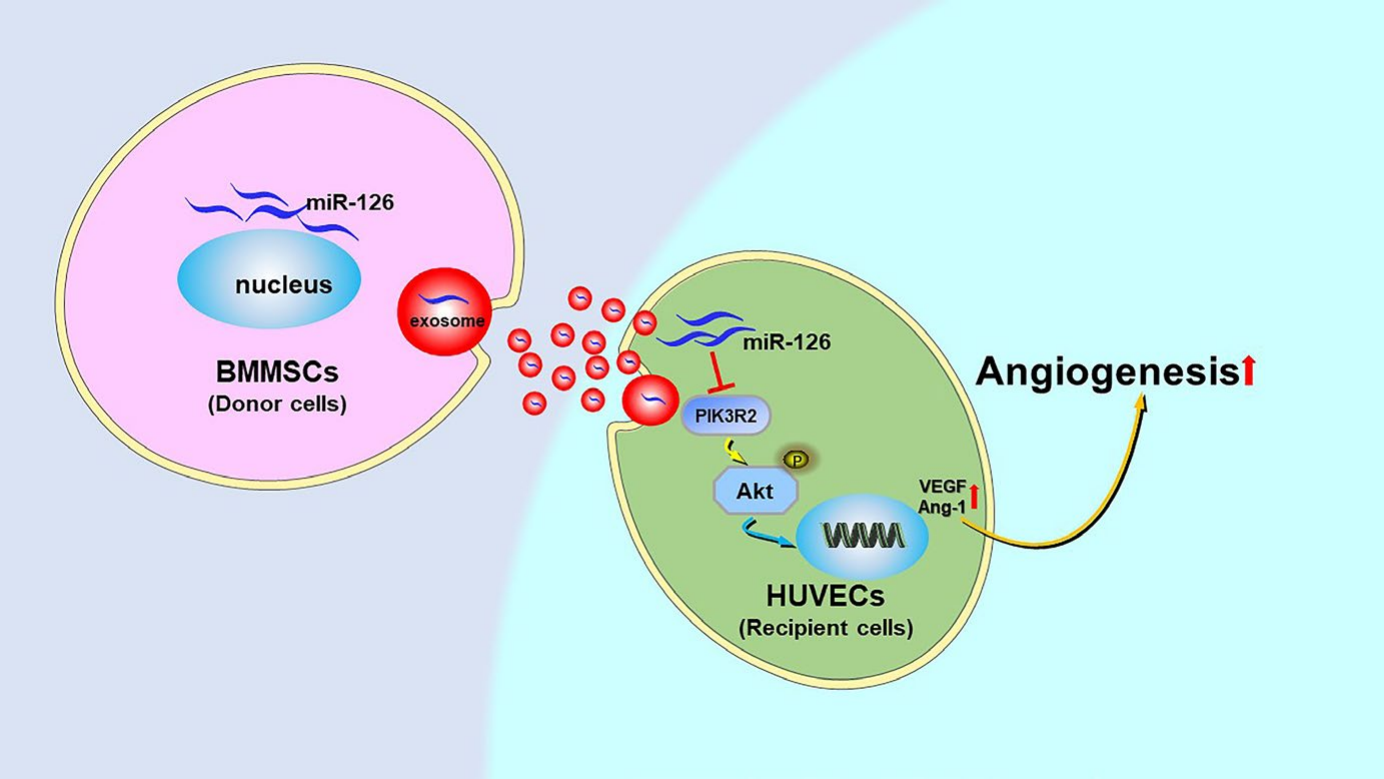
(Graphical abstract) Exosomes secreted from BMMSCs carrying miR‐126 promoted the angiogenesis of HUVECs by down‐regulating PIK3R2 to activate the PI3K/Akt signalling pathway. BMMSCs, bone marrow mesenchymal stem cells; HUVECs, human umbilical vein endothelial cells; PIK3R2, phosphoinositol‐3 kinase regulatory subunit 2

Then, the molecular mechanisms by which miR‐126 promote angiogenesis were explored. We focused on PIK3R2, the potential target of miR‐126, which plays a critical role in the PI3K/Akt pathway.^49^ PIK3R2 was down‐regulated by miR‐126 to activate the VEGF pathway to facilitate angiogenesis after injury.^12^ Also, Sessa et al., found that miR‐126 can regulate PIK3R2 to enhance the expression of Ang‐1, thus fine‐tuning vessel stabilization and its maturation.^38^ A previous study indicated that the pro‐angiogenic potential of exosomes derived from human placenta‐derived MSCs by nitric oxide stimulation was mainly mediated by miR‐126, which can regulate the PI3K/Akt pathway.^34^ Correspondingly, our present study demonstrated that after incubation with Exo‐miR‐126, the expression level of PIK3R2 was down‐regulated, while the level of Akt phosphorylation was up‐regulated in HUVECs. PIK3R2 was reported to inhibit the angiogenesis of endothelial cells by suppressing growth factor signalling via the PI3K/Akt pathway.^50^ In our present study, to further verify the effect of PIK3R2 on HUVECs, cell proliferation, migration and angiogenesis were detected by silencing PIK3R2 in HUVECs. The results demonstrated that the proliferation, migration and angiogenesis of HUVECs in the si‐PIK3R2 group were significantly increased relative to those in the si‐NC group. These results collectively suggested that Exo‐miR‐126 can promote angiogenesis by suppressing PIK3R2 to activate the PI3K/Akt signalling pathway in HUVECs (Figure 7). In a recent study, exosomes derived from MSCs loaded with miR‐126 accelerated angiogenesis and neurogenesis, inhibited apoptosis and promoted functional recovery after spinal cord injury, which was consistent with the findings of our current study.^17^ Additionally, in line with our findings, exosomes secreted by CD34+ peripheral blood mononuclear cells were demonstrated to deliver miR‐126 to endothelial cells to facilitate angiogenesis.^51^ Finally, a valid mouse model of skin defect was established to interrogate the pro‐angiogenic potential of Exo‐miR‐126,^20^, ^32^ and as expected, the local administration of miR‐126 modified exosomes accelerated wound healing by promoting newly formed capillaries.

Currently, a number of pro‐angiogenic miRNAs have been identified to affect neovascularization, however, the delivery of such miRNAs to target tissues to promote blood vessel formation remains challenging. Recent delivery configurations using exosomes and miRNAs have provided new insights for tissue regeneration, including angiogenesis. miRNAs such as miR‐126 can regulate a broad range of ‘natural’ mRNA targets and may therefore prove more efficient for fine‐tuning angiogenesis. Thus, a series of *in vitro* and *in vivo* studies were conducted to test and verify our previous hypothesis, and the results obtained from the current study indicate that exosomes derived from miR‐126 overexpressing BMMSCs represent a promising strategy in the promotion of angiogenesis related to regenerative medicine. More importantly, manipulation of miR‐126 levels in exosomes may prove therapeutic in a vast number of tissue regeneration scenarios (such as bone regeneration) of which angiogenesis is a critical component.

## 5 CONCLUSION

Our encouraging results showed that exosomes derived from miR‐126 overexpressing BMMSCs promoted the proliferation, migration and angiogenesis of HUVECs by regulating PI3K/Akt signalling pathway, and accelerated revascularization and wound healing *in vivo*. These provided evidence suggest that BMMSC‐derived exosomes combined with miR‐126 appear to be an appealing strategy for therapeutic angiogenesis and carry potential therapeutic significance. More importantly, the key findings of present study stretch our current understanding of exosomes, indicating that exosomes hold promise as vectors of therapeutic agents for the treatment of related diseases.

## CONFLICT OF INTEREST

The authors confirm that there are no conflicts of interest.

## AUTHOR CONTRIBUTION

Xijing He: Funding acquisition (supporting); Investigation (equal); Project administration (supporting); Supervision (supporting); Writing‐review & editing (equal). Lei Zhang: Conceptualization (lead); Methodology (lead); Writing‐original draft (lead). Pengrong Ouyang: Conceptualization (equal); Methodology (equal); Writing‐original draft (equal). Gaole He: Data curation (equal); Formal analysis (equal). Xiaowei Wang: Data curation (equal); Formal analysis (equal). Defu Song: Data curation (equal); Writing‐original draft (equal). Yijun Yang: Resources (equal); Supervision (equal); Writing‐review & editing (equal). Lei Zhang and Pengrong Ouyang: Study design; research performance and paper writing. Gaole He and Xiaowei Wang: Data analyses. Defu Song: Contribution to drafting the manuscript. Yijun Yang: Manuscript revision. Xijing He: Supervision of the experiments; manuscript revision; and approval of manuscript. All authors: Approval of final submitted manuscript.

## Supporting information

Fig S1

Fig S2

## Data Availability

The data that support the findings of this study are available from the corresponding author upon reasonable request.
