# Supplementary material for: Exosomes from microRNA‐126 overexpressing mesenchymal stem cells promote angiogenesis by targeting the PIK3R2‐mediated PI3K/Akt signalling pathway
Source: J Cell Mol Med. 2020 Dec 21;25(4):2148–62. doi: 10.1111/jcmm.16192 (PMC7882955; doi:10.1111/jcmm.16192)
Supplement: Supplementary file 1 — Fig S1 [file JCMM-25-2148-s001.docx]

**Supplemental material**


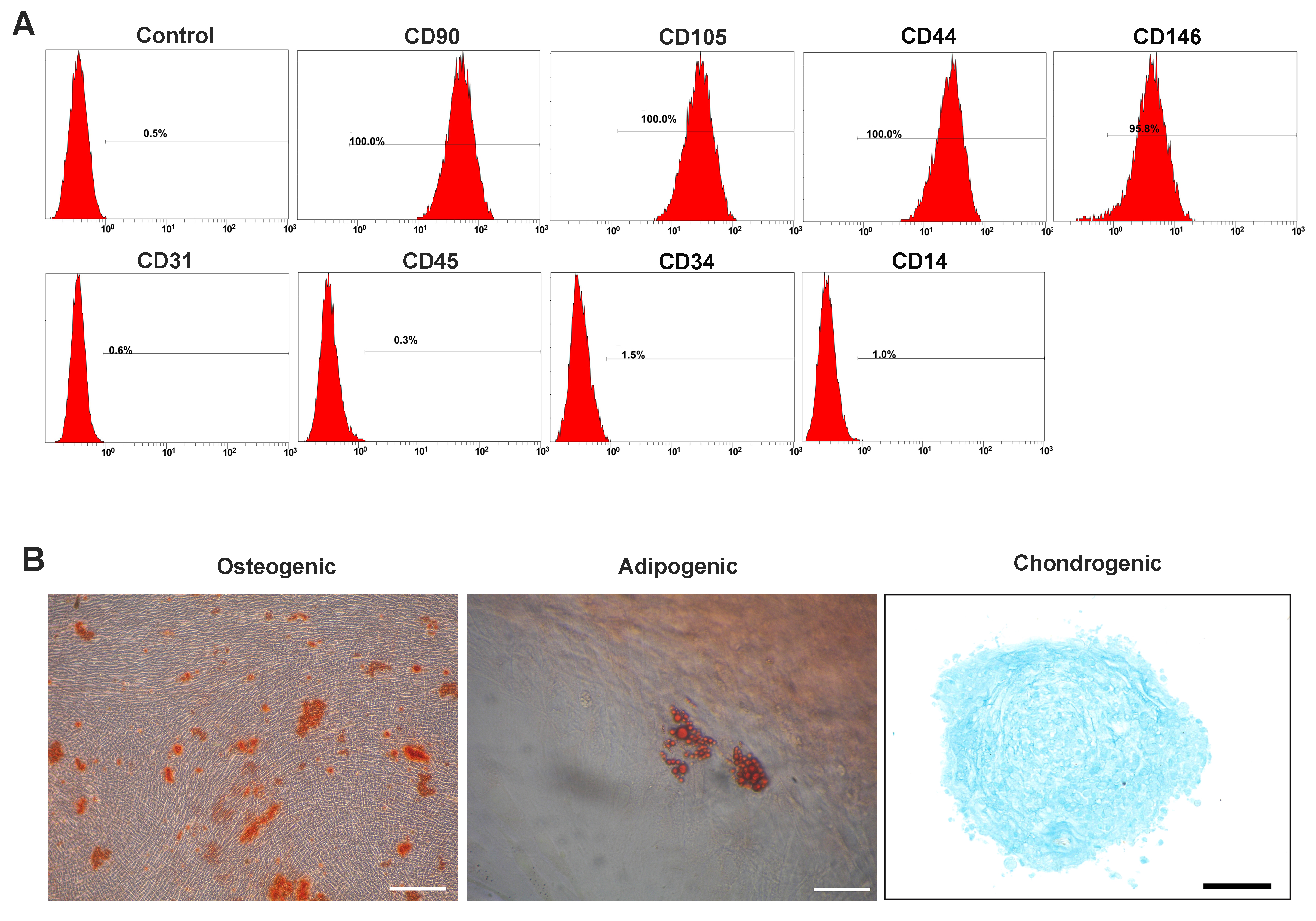


**Fig. S1. Identification of BMMSCs.** (A) Surface markers of BMMSCs assayed by flow cytometry: positive for CD90, CD105 CD44 and CD146, while negative for CD31, CD45, CD34 and CD14. (B) Multiple differentiation potentials of BMMSCs: Alizarin Red S staining for osteogenic differentiation (left; scale bar: 200 μm); Oil Red O staining for adipogenic differentiation (middle; scale bar: 200 μm); Alcian blue staining for chondrogenic differentiation (right; scale bar: 200 μm).
