# Supplementary material for: Exosomes from microRNA‐126 overexpressing mesenchymal stem cells promote angiogenesis by targeting the PIK3R2‐mediated PI3K/Akt signalling pathway
Source: J Cell Mol Med. 2020 Dec 21;25(4):2148–62. doi: 10.1111/jcmm.16192 (PMC7882955; doi:10.1111/jcmm.16192)
Supplement: Supplementary file 2 — Fig S2 [file JCMM-25-2148-s002.docx]

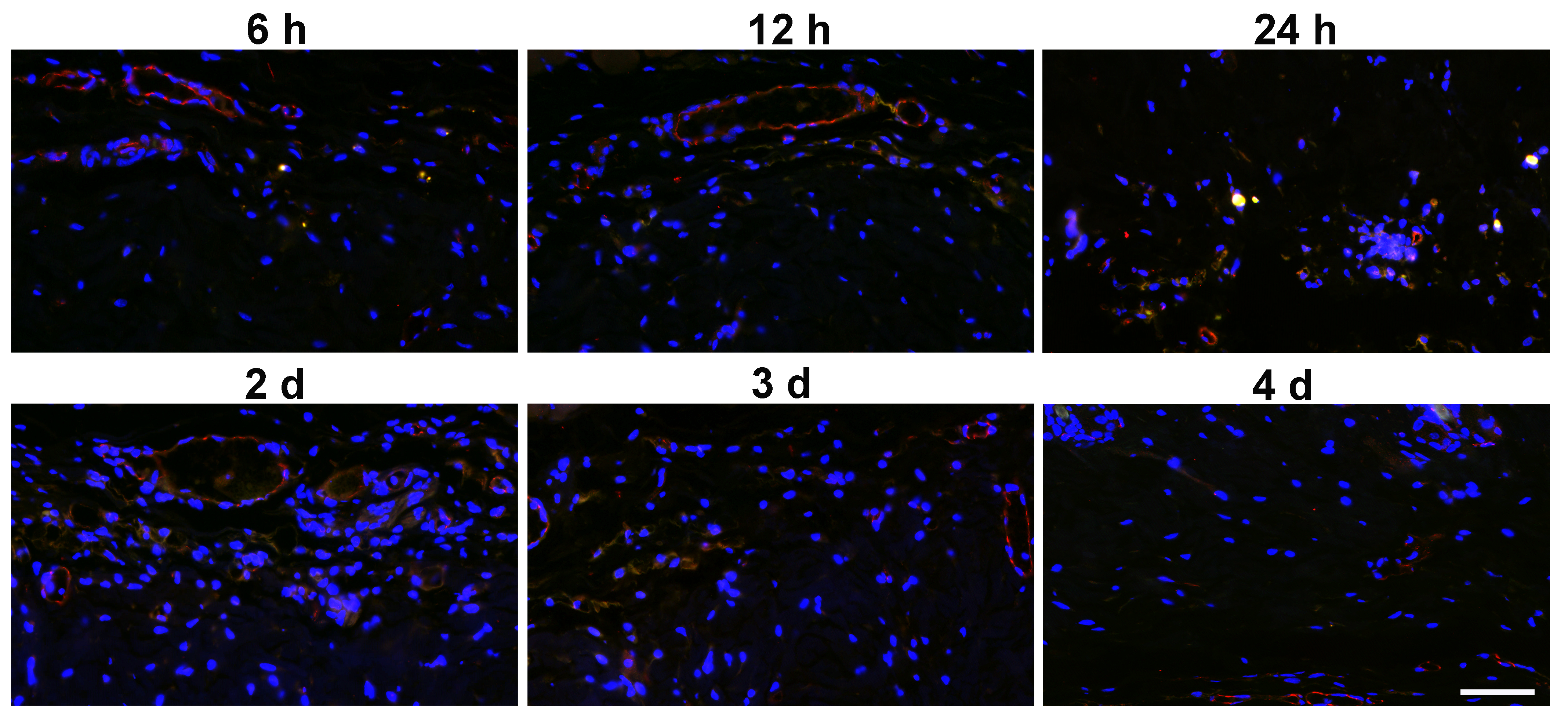


**Fig. S2.** **Retention of BMMSC-exosomes in skin tissues.** (A) Representative images of

BMMSC-Exos incorporation in skin tissues at different time point (Scale bar: 100 μm). To trace the administered BMMSC-Exos *in vivo*, exosomes were labeled with PKH67 and were subcutaneously injected into wound area at four different sites. 6 h, 12 h, 24 h, 2 d, 3 d or 4 d post-operation, the skin specimens were harvested, tissues were embedded in OCT and then cut into 10 μm thick sections, afterwards, nuclei were stained with DAPI, CD31 (red) were used to identify the endothelial cells. Images were observed with a fluorescence microscope.
